# Supplementary material for: SARS pandemic exposure impaired early childhood development in China
Source: Sci Rep. 2021 Apr 22;11:8694. doi: 10.1038/s41598-021-87875-8 (PMC8062548; doi:10.1038/s41598-021-87875-8)
Supplement: Supplementary file 1 — Supplementary Tables. [file 41598_2021_87875_MOESM1_ESM.doc]

**Supplemental materials** **of**

SARS pandemic exposure impaired early childhood development in China

Yunfei Fan^1, #^, Huiyu Wang^2, #^, Qiong Wu^3^, Xiang Zhou^4^, Yubo Zhou^2^, Bin Wang^2^, Yiqun Han^5^, Tao Xue^2, *^, Tong Zhu^1^

1. BIC-ESAT and SKL-ESPC, College of Environmental Science and Engineering, Peking University, Beijing, China;
2. Institute of Reproductive and Child Health / Ministry of Health Key Laboratory of Reproductive Health and Department of Epidemiology and Biostatistics, School of Public Health, Peking University, Beijing, China;
3. Institute of Social Science Survey, Peking University, Beijing, China;
4. College of Education, Purdue University, West Lafayette, Indiana, United States;
5. Environmental Research Group, MRC Centre for Environment and Health, Imperial College London, London, UK

* Corresponding to Dr. Tao Xue; Peking University Health Science Center, Xueyuan Road #38, Beijing, China; xuetaogk_9032@126.com

# Yunfei Fan and Huiyu Wang equally contribute to this manuscript.

Table S1 Associations between SARS and delayed milestones, estimated by different models.

| Milestone | Exposure | Hazard ratio (95% confidence intervals) of developmental delay | | |
| --- | --- | --- | --- | --- |
|  |  | Model 1* | Model 2** | Model 3*** |
| Walking independently | SARS_maternal_ | 0.946 (0.780, 1.148) | 0.967 (0.798, 1.174) | 1.011 (0.832, 1.229) |
|  | SARS_child_ | **3.343 (2.863, 3.903)** | **3.323 (2.844, 3.882)** | **3.168 (2.710, 3.703)** |
| Saying a complete sentence | SARS_maternal_ | 0.900 (0.743, 1.091) | 0.906 (0.748, 1.099) | 0.905 (0.746, 1.098) |
|  | SARS_child_ | **4.066 (3.571, 4.629)** | **4.038 (3.548, 4.597)** | **3.984 (3.500, 4.534)** |
| Counting from 1 to 10 | SARS_maternal_ | 1.021 (0.837, 1.245) | 1.022 (0.838, 1.246) | 1.029 (0.843, 1.256) |
|  | SARS_child_ | **5.207 (4.705, 5.763)** | **5.018 (4.534, 5.554)** | **4.960 (4.481, 5.489)** |
| Undressing him/herself for urination | SARS_maternal_ | 0.953 (0.782, 1.161) | 0.953 (0.781, 1.161) | 0.967 (0.792, 1.180) |
|  | SARS_child_ | **5.685 (5.103, 6.332)** | **5.600 (5.028, 6.237)** | **5.569 (5.000, 6.203)** |

* Model 1: adjustments of temporal trend and spatial random effect;

** Model 2: model 1 + adjustments of sex, ethnicity, and residence;

*** Model 3 (fully-adjusted model): model 2 + adjustments of gestational length, birthweight and breastfeeding duration.

The bolded estimates denote statistically significant associations.

Table S2 The estimated associations between the SARS pandemic and delayed milestones, with Bonferroni-corrected 95% confidence intervals.

|  | Exposure | Without mutual adjustment | With mutual adjustment |
| --- | --- | --- | --- |
|  |  | Hazard ratio | |
| Delayed milestones* | |  |  |
| Walking  independently | SARS_maternal_ | 1.011 (0.770, 1.327) | 1.390 (0.996, 1.940) |
|  | SARS_child_ | **3.168 (2.548, 3.939)** | **3.960 (3.103, 5.054)** |
| Saying a  complete sentence | SARS_maternal_ | 0.905 (0.691, 1.185) | **1.422 (1.018, 1.987)** |
|  | SARS_child_ | **3.984 (3.326, 4.772)** | **5.219 (4.285, 6.357)** |
| Counting  from 1 to 10 | SARS_maternal_ | 1.029 (0.779, 1.359) | **1.761 (1.250, 2.481)** |
|  | SARS_child_ | **4.960 (4.305, 5.714)** | **6.099 (5.244, 7.093)** |
| Undressing  him/herself for urination | SARS_maternal_ | 0.967 (0.732, 1.277) | **1.681 (1.196, 2.362)** |
|  | SARS_child_ | **5.569 (4.791, 6.**473) | **6.973 (5.927, 8.203)** |

The bolded estimates denote statistically significant associations.

Table S3 Associations between SARS and delayed milestones, estimated by different subgroups.

| Variable | Sub-group | Estimated effect (HR with 95% CI) of SARS_child_ by subpopulations | | | | | | | |
| --- | --- | --- | --- | --- | --- | --- | --- | --- | --- |
|  |  | Walking independently | | Saying a complete sentence | | Counting from 1 to 10 | | Undressing him/herself for urination | |
|  |  | HR(95%CI) | P | HR (95% CI) | P | HR (95%CI) | P | HR (95%CI) | P |
| Pandemic hotspot | Yes | 3.921(3.077,4.997) | 0.032 | 4.359(3.566,5.328) | 0.423 | 5.894(4.994,6.956) | 0.295 | 6.317(5.328,7.491) | 0.133 |
|  | No | 3.145(2.683,3.687) |  | 4.009(3.517,4.570) |  | 5.445(4.888,6.065) |  | 5.641(5.049,6.302) |  |
| Residence | Urban | 2.926(2.431,3.523) | 0.050 | 3.681(3.167,4.279) | 0.019 | 5.201(4.585,5.899) | 0.105 | 5.410(4.761,6.147) | 0.096 |
|  | Rural | 3.368(2.864,3.960) |  | 4.283(3.738,4.907) |  | 5.696(5.092,6.373) |  | 5.939(5.290,6.668) |  |
| Ethnicity | Han | 3.182(2.548,3.974) | 0.819 | 4.133(3.378,5.057) | 0.797 | 6.243(5.262,7.406) | 0.072 | 4.682(3.922,5.589) | 0.005 |
|  | Not Han | 3.247(2.771,3.805) |  | 4.050(3.559,4.610) |  | 5.421(4.873,6.031) |  | 5.880(5.268,6.564) |  |
| Preterm birth (≤ 37 weeks) | Yes | 2.978(2.087,4.251) | 0.778 | 4.291(3.085,5.968) | 0.757 | 4.813(3.607,6.421) | 0.461 | 6.321(4.781,8.357) | 0.852 |
|  | No | 3.130(2.672,3.667) |  | 4.487(3.922,5.132) |  | 5.360(4.826,5.952) |  | 6.152(5.503,6.878) |  |
| Low birthweight (< 2.5 kg) | Yes | 2.900(2.032,4.138) | 0.885 | 4.918(3.684,6.564) | 0.278 | 5.329(4.207,6.751) | 0.994 | 6.819(5.321,8.739) | 0.392 |
|  | No | 2.817(2.346,3.382) |  | 4.255(3.652,4.959) |  | 5.319(4.716,5.998) |  | 6.155(5.419,6.992) |  |
| Breast feeding | Yes | 3.223(2.754,3.772) | 0.608 | 4.034(3.545,4.591) | 0.519 | 5.479(4.929,6.090) | 0.273 | 5.691(5.104,6.346) | 0.123 |
|  | No | 3.463(2.589,4.633) |  | 4.361(3.420,5.562) |  | 6.073(4.932,7.477) |  | 6.589(5.357,8.104) |  |
| Sex | Male | 3.239(2.743,3.824) | 0.967 | 3.907(3.397,4.494) | 0.186 | 5.376(4.791,6.032) | 0.280 | 5.627(4.995,6.338) | 0.421 |
|  | Female | 3.235(2.717,3.853) |  | 4.232(3.670,4.881) |  | 5.676(5.043,6.389) |  | 5.862(5.193,6.618) |  |
|  | | Estimated effect (HR with 95% CI) of SARSmaternal by subpopulations | | | | | | | |
| Pandemic hotspot | Yes | 0.824(0.606,1.122) | 0.069 | 0.864(0.628,1.187) | 0.520 | 1.181(0.852,1.638) | 0.211 | 0.921(0.665,1.277) | 0.663 |
|  | No | 1.044(0.846,1.287) |  | 0.928(0.737,1.167) |  | 1.006(0.797,1.271) |  | 0.972(0.764,1.238) |  |
| Residence | Urban | 0.930(0.740,1.168) | 0.056 | 0.815(0.635,1.046) | 0.025 | 0.962(0.743,1.245) | 0.293 | 0.913(0.704,1.185) | 0.299 |
|  | Rural | 1.093(0.878,1.360) |  | 0.986(0.779,1.249) |  | 1.049(0.827,1.332) |  | 1.000(0.780,1.281) |  |
| Ethnicity | Han | 1.073(0.804,1.431) | 0.587 | 1.050(0.777,1.418) | 0.169 | 1.235(0.904,1.688) | 0.058 | 0.780(0.567,1.072) | 0.052 |
|  | Not Han | 1.016(0.823,1.255) |  | 0.892(0.707,1.126) |  | 0.984(0.777,1.245) |  | 0.998(0.784,1.272) |  |
| Preterm birth (≤ 37 weeks) | Yes | 1.274(0.817,1.984) | 0.320 | 0.856(0.551,1.330) | 0.809 | 1.109(0.713,1.723) | 0.707 | 0.824(0.523,1.299) | 0.400 |
|  | No | 1.048(0.834,1.319) |  | 0.894(0.711,1.124) |  | 1.030(0.815,1.300) |  | 0.962(0.750,1.234) |  |
| Low birthweight (<2.5 kg) | Yes | 1.486(0.925,2.389) | 0.064 | 1.256(0.791,1.995) | 0.086 | 1.171(0.736,1.863) | 0.372 | 0.981(0.603,1.597) | 0.602 |
|  | No | 1.031(0.804,1.324) |  | 0.902(0.703,1.156) |  | 0.980(0.758,1.266) |  | 0.884(0.677,1.155) |  |
| Breast feeding | Yes | 1.014(0.823,1.249) | 0.181 | 0.917(0.729,1.153) | 0.722 | 1.003(0.793,1.268) | 0.270 | 0.954(0.749,1.215) | 0.468 |
|  | No | 1.219(0.867,1.714) |  | 0.974(0.684,1.385) |  | 1.175(0.827,1.669) |  | 1.064(0.751,1.507) |  |
| Sex | Male | 0.959(0.768,1.199) | 0.102 | 0.993(0.780,1.263) | 0.053 | 1.085(0.849,1.386) | 0.123 | 0.995(0.772,1.282) | 0.485 |
|  | Female | 1.090(0.872,1.361) |  | 0.847(0.666,1.077) |  | 0.956(0.748,1.221) |  | 0.940(0.731,1.209) |  |

HR: hazard ratio; CI: confidence interval; P: p-value for the null-hypothesis that the estimated HRs are identical between subpopulations.

Table S4 The enhanced association between SARS and delayed milestone by continuous indicators for size of the pandemic.

| Outcome | Enhancement effect* by ***x*** (95% CI); P-value | |
| --- | --- | --- |
|  | ***x*** = number of deaths | ***x*** = number of cases |
| Walking independently | 0.46% (-0.02%, 0.94%); P=0.062 | 0.24% (-0.06%, 0.54%); P=0.114 |
| Saying a complete sentence | 0.36% (-0.06%, 0.79%); P=0.096 | 0.26% (-0.01%, 0.53%); P=0.061 |
| Counting from 1 to 10 | 0.31% (-0.05%, 0.68%); P=0.092 | 0.12% (-0.10%, 0.35%); P=0.282 |
| Undressing him/herself for urination | 0.45% (0.08%, 0.82%); P=0.016 | 0.32% (0.08%, 0.55%); P=0.008 |

* Enhancement effect: the increase in hazard ratio (%) per 10% increment in the number of SARS deaths or cases.

Table S5 The estimated association between the delayed milestones and alternative exposure indicators for the SARS pandemic during childhood.

|  | Association between the delayed milestone and SARS_child_ ^*^  (hazard ratio with 95% CI) | | |
| --- | --- | --- | --- |
| Exposure indicator | A binary indicator for exposure to the whole pandemic^#^ | A continuous indicator: increased risk for a month increment in duration of the pandemic exposure | |
| Walking  independently | **7.379 (6.538, 8.33)** | | **1.339 (1.318, 1.36)** |
| Saying  a complete sentence | **3.217 (2.724, 3.80)** | | **1.324 (1.281, 1.37)** |
| Counting  from 1 to 10 | **7.885 (6.535, 9.51)** | | **1.348 (1.317, 1.38)** |
| Undressing him/herself for urination | **9.377 (8.200, 10.72)** | | **1.374 (1.350, 1.40)** |

* The association was not mutually adjusted by SARS_maternal_;

# In the analyses, we excluded the samples who partially experienced the SARS pandemic.

The bolded estimates denote statistically significant associations.

Table S6 The estimated associations between SARS and body weight/height by different models.

| Exposure | Weight (kg) change with 95% confidence intervals | | | |
| --- | --- | --- | --- | --- |
|  | Model 1* | Model 2** | Model 3*** | Model 4**** |
| SARS_maternal_ | 1.13 (-0.55, 2.81) | 1.34 (-0.33, 3.02) | 1.48 (-0.17, 3.13) | 1.32 (-0.31, 2.95) |
| SARS_child, age 0_ | **-3.07 (-5.50, -0.65)** | -2.22 (-4.62, 0.18) | **-2.45 (-4.81, -0.08)** | **-2.45 (-4.79, -0.12)** |
| SARS_child, age 1_ | **-8.07 (-11.35, -4.78)** | **-4.69 (-7.89, -1.49)** | **-4.67 (-7.82, -1.53)** | **-4.41 (-7.51, -1.30)** |
| SARS_child, age 2_ | **-12.53 (-17.62, -7.44)** | **-6.73 (-11.63, -1.82)** | **-6.49 (-11.31, -1.68)** | **-5.99 (-10.75, -1.22)** |
| SARS_child, age 3_ | **-15.93 (-22.71, -9.14)** | **-7.91 (-14.43, -1.38)** | **-7.50 (-13.91, -1.09)** | **-6.83 (-13.17, -0.49)** |
| SARS_child, age 4_ | **-17.74 (-25.80, -9.68)** | **-7.80 (-15.55, -0.05)** | -7.30 (-14.92, 0.31) | -6.55 (-14.09, 0.98) |
| SARS_child, age 5_ | **-17.61 (-26.56, -8.66)** | -6.12 (-14.67, 2.43) | -5.62 (-14.02, 2.78) | -4.92 (-13.23, 3.39) |
| SARS_child, age 6_ | **-15.89 (-25.73, -6.04)** | -3.16 (-12.37, 6.06) | -2.72 (-11.78, 6.34) | -2.17 (-11.13, 6.79) |
| SARS_child, age 7_ | **-13.11 (-24.21, -2.00)** | 0.67 (-9.40, 10.73) | 0.99 (-8.91, 10.88) | 1.32 (-8.47, 11.11) |
| SARS_child, age 8_ | -9.79 (-22.65, 3.06) | 4.92 (-6.33, 16.17) | 5.10 (-5.97, 16.17) | 5.18 (-5.77, 16.13) |
|  | Height (cm) change with 95% confidence intervals | | | |
| SARS_maternal_ | -0.72 (-3.38, 1.93) | -0.05 (-2.68, 2.59) | 0.02 (-2.55, 2.58) | -0.08 (-2.61, 2.45) |
| SARS_child, age 0_ | 0.49 (-3.56, 4.54) | -0.48 (-4.47, 3.50) | -0.81 (-4.69, 3.07) | -0.97 (-4.80, 2.85) |
| SARS_child, age 1_ | -2.35 (-7.88, 3.19) | -0.34 (-5.62, 4.95) | -0.54 (-5.67, 4.60) | -0.35 (-5.42, 4.72) |
| SARS_child, age 2_ | -5.08 (-13.73, 3.56) | -0.23 (-8.31, 7.86) | -0.31 (-8.18, 7.55) | 0.16 (-7.60, 7.93) |
| SARS_child, age 3_ | -7.62 (-19.16, 3.92) | -0.18 (-10.93, 10.58) | -0.20 (-10.66, 10.26) | 0.47 (-9.85, 10.80) |
| SARS_child, age 4_ | -9.86 (-23.56, 3.84) | -0.22 (-12.97, 12.54) | -0.25 (-12.66, 12.16) | 0.47 (-11.78, 12.73) |
| SARS_child, age 5_ | -11.73 (-27.00, 3.54) | -0.37 (-14.43, 13.69) | -0.50 (-14.18, 13.18) | 0.10 (-13.41, 13.60) |
| SARS_child, age 6_ | -13.31 (-30.30, 3.69) | -0.61 (-15.78, 14.55) | -0.91 (-15.67, 13.86) | -0.59 (-15.17, 13.99) |
| SARS_child, age 7_ | -14.68 (-34.22, 4.86) | -0.92 (-17.54, 15.70) | -1.42 (-17.62, 14.77) | -1.49 (-17.48, 14.51) |
| SARS_child, age 8_ | -15.96 (-38.99, 7.07) | -1.26 (-19.94, 17.42) | -1.99 (-20.21, 16.22) | -2.48 (-20.49, 15.52) |

* Model 1: the independent variables include SARS_maternal_, SARS_child_, temporal trend and spatial random effect;

** Model 2: model 1 + adjustments of nonlinear effect of age;

*** Model 3: model 2 + adjustments of residence, sex and ethnicity;

**** Model 4: model 3 + adjustments of birthweight, gestational length and breastfeeding duration.

The bolded estimates denote statistically significant associations.

Table S7 The estimated associations between SARS and body weight/height by subpopulations.

|  | Weight change (kg) | | Height change (cm) | |
| --- | --- | --- | --- | --- |
|  | Pandemic hotspot | | Pandemic hotspot | |
|  | No | Yes | No | Yes |
| SARS_maternal_ | 1.29 (-0.35, 2.93) | 1.44 (-0.85, 3.73) | -0.22 (-2.77, 2.32) | 1.28 (-2.24, 4.80) |
| SARS_child, age 0_ | **-2.47 (-4.83, -0.12)** | -2.55 (-5.25, 0.15) | -1.08 (-4.90, 2.75) | -0.16 (-4.53, 4.20) |
| SARS_child, age 1_ | **-4.33 (-7.45, -1.22)** | **-5.02 (-8.24, -1.79)** | -0.36 (-5.43, 4.72) | -0.04 (-5.32, 5.25) |
| SARS_child, age 2_ | **-5.83 (-10.60, -1.07)** | **-7.05 (-11.86, -2.25)** | 0.25 (-7.52, 8.01) | 0.05 (-7.81, 7.91) |
| SARS_child, age 3_ | **-6.61 (-12.95, -0.27)** | **-8.24 (-14.62, -1.86)** | 0.62 (-9.71, 10.95) | 0.04 (-10.36, 10.45) |
| SARS_child, age 4_ | -6.31 (-13.84, 1.22) | **-8.15 (-15.72, -0.57)** | 0.64 (-11.62, 12.89) | -0.10 (-12.42, 12.23) |
| SARS_child, age 5_ | -4.69 (-12.99, 3.62) | -6.49 (-14.84, 1.85) | 0.23 (-13.28, 13.74) | -0.40 (-13.96, 13.16) |
| SARS_child, age 6_ | -1.98 (-10.94, 6.97) | -3.56 (-12.54, 5.43) | -0.52 (-15.11, 14.06) | -0.83 (-15.46, 13.80) |
| SARS_child, age 7_ | 1.44 (-8.35, 11.23) | 0.23 (-9.60, 10.06) | -1.51 (-17.51, 14.49) | -1.36 (-17.44, 14.72) |
| SARS_child, age 8_ | 5.22 (-5.73, 16.18) | 4.44 (-6.59, 15.47) | -2.62 (-20.63, 15.39) | -1.93 (-20.09, 16.23) |
|  | Residence | | Residence | |
|  | Rural | Urban | Rural | Urban |
| SARS_maternal_ | 0.60 (-1.09, 2.29) | 1.98 (0.31, 3.65) | -0.65 (-3.33, 2.03) | 0.39 (-2.23, 3.01) |
| SARS_child, age 0_ | **-3.11 (-5.68, -0.55)** | **-2.65 (-4.99, -0.30)** | -1.18 (-5.06, 2.69) | -1.63 (-5.96, 2.69) |
| SARS_child, age 1_ | **-5.81 (-8.99, -2.63)** | **-4.77 (-7.87, -1.67)** | -0.67 (-5.76, 4.42) | -1.33 (-6.59, 3.93) |
| SARS_child, age 2_ | **-8.12 (-12.89, -3.34)** | **-6.53 (-11.26, -1.79)** | -0.24 (-8.01, 7.53) | -1.07 (-8.93, 6.79) |
| SARS_child, age 3_ | **-9.65 (-15.98, -3.31)** | **-7.56 (-13.85, -1.26)** | 0.02 (-10.32, 10.35) | -0.90 (-11.32, 9.51) |
| SARS_child, age 4_ | **-10.01 (-17.53, -2.50)** | **-7.49 (-14.97, -0.01)** | 0.02 (-12.24, 12.28) | -0.88 (-13.21, 11.46) |
| SARS_child, age 5_ | **-8.96 (-17.24, -0.67)** | -6.09 (-14.34, 2.17) | -0.29 (-13.80, 13.22) | -1.02 (-14.59, 12.55) |
| SARS_child, age 6_ | -6.74 (-15.67, 2.20) | -3.59 (-12.50, 5.31) | -0.86 (-15.44, 13.73) | -1.30 (-15.95, 13.35) |
| SARS_child, age 7_ | -3.74 (-13.54, 6.05) | -0.37 (-10.10, 9.37) | -1.59 (-17.60, 14.41) | -1.68 (-17.80, 14.44) |
| SARS_child, age 8_ | -0.36 (-11.37, 10.65) | 3.22 (-7.67, 14.12) | -2.42 (-20.45, 15.61) | -2.10 (-20.34, 16.14) |
|  | Ethnicity | | Ethnicity | |
|  | Han | Not Han | Han | Not Han |
| SARS_maternal_ | 1.44 (-0.21, 3.08) | 1.14 (-1.04, 3.32) | -0.04 (-2.61, 2.52) | 0.78 (-2.70, 4.27) |
| SARS_child, age 0_ | -1.32 (-4.11, 1.47) | **-2.34 (-4.68, -0.00)** | 2.55 (-2.14, 7.25) | -0.60 (-4.43, 3.24) |
| SARS_child, age 1_ | -2.03 (-5.30, 1.24) | **-4.14 (-7.24, -1.04)** | 2.72 (-2.69, 8.12) | -0.08 (-5.15, 4.99) |
| SARS_child, age 2_ | -2.50 (-7.34, 2.34) | **-5.57 (-10.32, -0.82)** | 2.89 (-5.03, 10.82) | 0.35 (-7.41, 8.11) |
| SARS_child, age 3_ | -2.47 (-8.90, 3.95) | -6.28 (-12.60, 0.04) | 3.10 (-7.38, 13.57) | 0.61 (-9.70, 10.92) |
| SARS_child, age 4_ | -1.71 (-9.32, 5.90) | -5.90 (-13.41, 1.61) | 3.34 (-9.04, 15.73) | 0.62 (-11.62, 12.85) |
| SARS_child, age 5_ | -0.04 (-8.41, 8.32) | -4.18 (-12.47, 4.10) | 3.64 (-9.96, 17.24) | 0.31 (-13.17, 13.80) |
| SARS_child, age 6_ | 2.36 (-6.64, 11.37) | -1.37 (-10.31, 7.56) | 3.98 (-10.69, 18.65) | -0.25 (-14.81, 14.32) |
| SARS_child, age 7_ | 5.26 (-4.60, 15.13) | 2.16 (-7.60, 11.92) | 4.35 (-11.81, 20.51) | -0.97 (-16.95, 15.00) |
| SARS_child, age 8_ | 8.41 (-2.71, 19.52) | 6.06 (-4.86, 16.98) | 4.73 (-13.61, 23.08) | -1.79 (-19.77, 16.20) |
|  | Sex | | Sex | |
|  | Female | Male | Female | Male |
| SARS_maternal_ | 1.19 (-0.51, 2.89) | 1.56 (-0.17, 3.29) | 0.77 (-1.91, 3.45) | -0.88 (-3.55, 1.79) |
| SARS_child, age 0_ | **-4.51 (-7.23, -1.78)** | **-3.16 (-5.53, -0.78)** | -1.98 (-6.46, 2.50) | -1.15 (-5.05, 2.74) |
| SARS_child, age 1_ | **-6.40 (-9.62, -3.18)** | **-5.03 (-8.14, -1.92)** | -0.96 (-6.23, 4.32) | -0.38 (-5.46, 4.70) |
| SARS_child, age 2_ | **-7.97 (-12.78, -3.16)** | **-6.56 (-11.32, -1.80)** | -0.23 (-8.09, 7.63) | 0.23 (-7.53, 8.00) |
| SARS_child, age 3_ | **-8.91 (-15.32, -2.51)** | **-7.40 (-13.74, -1.06)** | -0.08 (-10.52, 10.35) | 0.51 (-9.82, 10.83) |
| SARS_child, age 4_ | **-8.91 (-16.51, -1.31)** | -7.19 (-14.73, 0.34) | -0.81 (-13.18, 11.55) | 0.27 (-11.98, 12.53) |
| SARS_child, age 5_ | -7.75 (-16.12, 0.61) | -5.72 (-14.03, 2.58) | -2.61 (-16.20, 10.98) | -0.58 (-14.08, 12.92) |
| SARS_child, age 6_ | -5.65 (-14.65, 3.34) | -3.21 (-12.16, 5.74) | -5.29 (-19.93, 9.35) | -1.94 (-16.51, 12.63) |
| SARS_child, age 7_ | -2.92 (-12.75, 6.91) | -0.01 (-9.78, 9.77) | -8.55 (-24.64, 7.54) | -3.64 (-19.62, 12.34) |
| SARS_child, age 8_ | 0.13 (-10.91, 11.17) | 3.54 (-7.39, 14.48) | -12.10 (-30.29, 6.09) | -5.50 (-23.49, 12.49) |
|  | Preterm birth | | Preterm birth | |
|  | No | Yes | No | Yes |
| SARS_maternal_ | 1.69 (-0.05, 3.43) | 1.16 (-0.56, 2.87) | -0.01 (-2.72, 2.71) | 0.04 (-2.64, 2.71) |
| SARS_child, age 0_ | -2.22 (-5.02, 0.57) | **-2.41 (-4.82, -0.01)** | 0.56 (-4.02, 5.14) | -0.41 (-4.35, 3.53) |
| SARS_child, age 1_ | **-3.81 (-7.09, -0.54)** | **-4.23 (-7.37, -1.10)** | 0.01 (-5.36, 5.38) | -0.18 (-5.30, 4.93) |
| SARS_child, age 2_ | **-5.05 (-9.89, -0.22)** | **-5.69 (-10.46, -0.93)** | -0.47 (-8.37, 7.43) | -0.00 (-7.79, 7.78) |
| SARS_child, age 3_ | -5.59 (-12.00, 0.81) | **-6.44 (-12.78, -0.09)** | -0.81 (-11.24, 9.63) | 0.09 (-10.25, 10.43) |
| SARS_child, age 4_ | -5.09 (-12.68, 2.50) | -6.10 (-13.64, 1.43) | -0.93 (-13.28, 11.42) | 0.05 (-12.21, 12.31) |
| SARS_child, age 5_ | -3.30 (-11.65, 5.05) | -4.46 (-12.77, 3.85) | -0.80 (-14.38, 12.77) | -0.15 (-13.66, 13.36) |
| SARS_child, age 6_ | -0.47 (-9.46, 8.52) | -1.73 (-10.69, 7.23) | -0.46 (-15.10, 14.18) | -0.48 (-15.06, 14.11) |
| SARS_child, age 7_ | 3.05 (-6.79, 12.90) | 1.71 (-8.07, 11.50) | 0.02 (-16.10, 16.14) | -0.90 (-16.91, 15.11) |
| SARS_child, age 8_ | 6.93 (-4.14, 18.00) | 5.52 (-5.44, 16.47) | 0.57 (-17.69, 18.83) | -1.36 (-19.39, 16.68) |
|  | Low birthweight | | Low birthweight | |
|  | No | Yes | No | Yes |
| SARS_maternal_ | 1.37 (-0.26, 3.00) | -0.80 (-3.39, 1.78) | -0.13 (-2.66, 2.40) | 3.26 (-1.84, 8.37) |
| SARS_child, age 0_ | **-3.62 (-6.54, -0.70)** | **-2.53 (-4.86, -0.19)** | -2.54 (-7.34, 2.25) | -0.91 (-4.74, 2.91) |
| SARS_child, age 1_ | **-4.88 (-8.20, -1.56)** | **-4.43 (-7.54, -1.32)** | -1.87 (-7.25, 3.51) | -0.24 (-5.31, 4.84) |
| SARS_child, age 2_ | **-5.82 (-10.67, -0.96)** | **-5.97 (-10.74, -1.21)** | -1.26 (-9.17, 6.64) | 0.33 (-7.44, 8.10) |
| SARS_child, age 3_ | -6.12 (-12.54, 0.31) | **-6.78 (-13.12, -0.44)** | -0.79 (-11.27, 9.70) | 0.68 (-9.66, 11.02) |
| SARS_child, age 4_ | -5.46 (-13.08, 2.15) | -6.49 (-14.02, 1.05) | -0.50 (-12.93, 11.92) | 0.71 (-11.56, 12.97) |
| SARS_child, age 5_ | -3.64 (-12.02, 4.73) | -4.85 (-13.16, 3.46) | -0.46 (-14.10, 13.19) | 0.34 (-13.18, 13.85) |
| SARS_child, age 6_ | -0.87 (-9.88, 8.15) | -2.11 (-11.07, 6.85) | -0.60 (-15.31, 14.10) | -0.36 (-14.95, 14.23) |
| SARS_child, age 7_ | 2.55 (-7.32, 12.42) | 1.36 (-8.43, 11.15) | -0.88 (-17.06, 15.31) | -1.28 (-17.28, 14.73) |
| SARS_child, age 8_ | 6.28 (-4.84, 17.41) | 5.20 (-5.76, 16.15) | -1.22 (-19.59, 17.15) | -2.30 (-20.31, 15.71) |
|  | Breastfeeding | | Breastfeeding | |
|  | No | Yes | No | Yes |
| SARS_maternal_ | 2.10 (-0.23, 4.44) | 1.28 (-0.36, 2.92) | -1.30 (-5.09, 2.49) | 0.03 (-2.51, 2.56) |
| SARS_child, age 0_ | -1.07 (-5.09, 2.95) | -1.82 (-4.62, 0.97) | 0.41 (-6.14, 6.96) | -0.31 (-4.88, 4.26) |
| SARS_child, age 1_ | -2.73 (-6.56, 1.09) | **-3.58 (-6.87, -0.28)** | 1.59 (-4.68, 7.87) | 0.65 (-4.73, 6.04) |
| SARS_child, age 2_ | -4.18 (-9.44, 1.09) | **-5.04 (-9.94, -0.14)** | 2.38 (-6.23, 10.99) | 1.36 (-6.64, 9.36) |
| SARS_child, age 3_ | -5.17 (-12.08, 1.75) | -5.92 (-12.41, 0.58) | 2.37 (-8.88, 13.63) | 1.55 (-9.03, 12.13) |
| SARS_child, age 4_ | -5.48 (-13.56, 2.61) | -5.92 (-13.60, 1.76) | 1.18 (-11.95, 14.30) | 0.95 (-11.53, 13.44) |
| SARS_child, age 5_ | -4.96 (-13.67, 3.76) | -4.84 (-13.26, 3.58) | -1.47 (-15.62, 12.68) | -0.60 (-14.28, 13.08) |
| SARS_child, age 6_ | -3.76 (-13.10, 5.58) | -2.89 (-11.96, 6.17) | -5.31 (-20.55, 9.93) | -2.93 (-17.69, 11.84) |
| SARS_child, age 7_ | -2.11 (-12.63, 8.40) | -0.36 (-10.35, 9.63) | -9.94 (-27.27, 7.40) | -5.78 (-22.15, 10.59) |
| SARS_child, age 8_ | -0.24 (-12.66, 12.19) | 2.47 (-8.89, 13.83) | -14.96 (-35.63, 5.71) | -8.90 (-27.65, 9.85) |

The bolded estimates denote statistically significant associations.
